# Effects of poor hygiene on cytokine phenotypes in children in the tropics

**DOI:** 10.1186/s40413-016-0124-1

**Published:** 2016-11-03

**Authors:** C. A. Figueiredo, L. D. Amorim, M. Vaca, M. E. Chico, A. C. Campos, M. L. Barreto, P. J. Cooper

**Affiliations:** 1 Instituto de Ciencias da Saude, Universidade Federal da Bahia, Salvador, Brazil; 2 Instituto de Matematica, Universidade Federal da Bahia, Salvador, Brazil; 3 Laboratorio de Investigaciones FEPIS, Quininde, Esmeraldas Province Ecuador; 4 Instituto de Saúde Coletiva, Universidade Federal da Bahia, Salvador, Brazil; 5 Faculty of Facultad de Ciencias Medicas, de la Salud y la Vida, Universidad Internacional del Ecuador, Casilla 17-22-20418, Quito, Ecuador; 6 Institute of Infection and Immunity, St George’s University of London, London, UK

**Keywords:** Cytokines, Environment, Hygiene, Latent class analysis, Tropics, Latin America

## Abstract

We describe immune phenotypes (innate and adaptive cytokines) according to environmental exposure using latent class analysis. A total of 310 schoolchildren living in Ecuador were assayed for spontaneous cytokine production as well as mitogen (SEB)-stimulated cytokines in whole blood cultures. We collected data on environmental exposures by questionnaire and on intestinal parasites by examination of stool samples. Latent class analysis (LCA) was used to group children according to their innate (IL-6, IL-8, IL-10 and TNF-α) and adaptive (IL-5, IL-13, IL-17, IFN-γ and IL-10) cytokine profile. We also conducted multiple-group LCA and LCA with covariates to evaluate the effect of predictors on profile membership. We identified both hyporesponsive and Th2-modified immune phenotypes produced by peripheral blood leukocytes (PBLs) that were associated with intestinal worms and birth order, providing insights into how poor hygiene mediates immunologic effects on immune-mediated diseases.

**Electronic supplementary material:**

The online version of this article (doi:10.1186/s40413-016-0124-1) contains supplementary material, which is available to authorized users.

**To the Editor:**

The hygiene hypothesis explains worldwide trends of increasing allergic diseases as being caused by improvements in hygiene and declines in exposures to microbes and infections leading to a failure in the development of robust immune regulation [1]. There are few data investigating how poor hygiene exposures may affect the human immune response in tropical settings. We have shown previously greater production of immune regulatory IL-10 by peripheral blood leukocytes (PBLs) in Latin American children with poor hygiene exposures [2–4]. In the present analysis, we extend these observations in Ecuadorian schoolchildren using latent class analysis to define immune phenotypes of innate and adaptive cytokines produced by PBLs at homeostasis and after maximal stimulation and show how poor hygiene exposures affect immune response phenotypes.

We analysed data from nested case–control studies of wheezing illness done in schoolchildren living in Esmeraldas Province, Ecuador as described previously [4]. Data on asthma symptoms, and demographic and environmental factor data were obtained by parental questionnaire, and stools were analyzed by microscopy for geohelminth parasites [4]. Skin prick tests (SPT) were done to 7 relevant aeroallergens including *Dermatophagoides pteronyssinus/farinae* and American cockroach [5]. Blood was collected into heparinized tubes and diluted 1:4 in RPMI 1640 (BioWhittaker, Walkersville, MD) with L-glutamine (BioWhittaker), 80 mg/ml gentamicin and 1 % HEPES (GIBCO, Gaithersburg, MD). PBLs were cultured alone for 24 (innate cytokines) hours and 5 days (adaptive cytokines) or with *Staphylococcus* enterotoxin B (SEB) (Sigma Aldrich, St Louis, MO, USA) at 1 μg/mL for 5 days to measure spontaneous and SEB-stimulated cytokines, respectively. Adaptive Th2 (IL[interleukin]-5 and IL-13), Th1 (Interferon-gamma [IFN-γ]), Th17 (IL-17) and Treg (IL-10) cytokines as well as pro-inflammatory cytokines IL-6, and TNF-α and the chemokine IL-8 of the innate immune response were measured in culture supernatants by sandwich ELISA (DuoSet, R&D Systems, Minneapolis, MN, USA). Because there were no significant differences in PBL cytokine responses between wheeze cases and non-wheeze control children, cytokine data for cases and controls were pooled. For spontaneous cytokine/chemokine production (i.e. homeostasis), responders were defined using the lower detection limits of the assays while for SEB-stimulation (i.e. maximal stimulation), we used median values for each cytokine/chemokine as cut-offs. Analyses were done using Latent Class Analysis (LCA) to identify phenotypes associated with each of homeostatic and maximal stimulation. For our analyses, outcomes were cytokine profile membership (i.e. defined *a posteriori*) with exposures being poor hygiene-related variables. LCA with covariates was used to include predictors of immune phenotype membership and log-likelihood tests to evaluate the overall effect of each covariate using MPlus (version 5). Informed written parental consent and minor assent was obtained for all children.

Demographic and environmental characteristics of 310 schoolchildren studied were (Additional file 1: Table S1): 53.2 % were rural, 45.5 % were girls, 89.4 % were Afro-Ecuadorian, 57.4 % were aged 6-10 years, 50.7 % lived in overcrowded houses (≥ median of 2.7 people/sleeping room), 62.6 % of fathers were involved in agriculture, 39 % of children were 4^th^ or greater in birth order, 35.8 % lived in houses ≥4 peridomiciliary animals, and half (50.7 %) of the children were infected with geohelminth parasites, predominantly *Ascaris lumbricoides* and *Trichuris trichiura*. We identified 2 immune phenotypes of cytokine/chemokine production for each of homeostatic and maximal immune responses (Table 1). For homeostasis, we identified low and high response phenotypes in which the former was characterized by lower overall detection frequencies for innate pro-inflammatory and adaptive cytokines (Table 1). For the maximal immune stimulus, we identified low and modified Th2 response phenotypes, the latter characterized by a higher frequency of the production of Th2 (IL-5 and IL-13) and IL-10 but similar frequencies of IL-17 and IFN-γ compared to low response phenotype (Table 1). When we evaluated the effects of poor hygiene exposures on phenotypes (Table 2 and Additional file 2: Figure S1), we observed that children infected with geohelminths and those higher in the birth order were more likely to have a low response phenotype at homeostasis and a modified Th2 response following a maximal immune stimulus. A modified Th2 response was less frequent among children whose fathers were engaged in agriculture (Table 2). The high response phenotype at homeostasis was negatively associated with SPT reactivity (adj. OR 0.43, 95 % CI 0.20-0.89) but immune phenotypes were not associated with asthma (data not shown).

**Table 1:** Proportions of children producing cytokines in spontaneous and SEB-stimulated peripheral blood leukocyte cultures by immunological phenotype

Cytokines	Overall % (N = 310)		
		Spontaneous immune phenotype	
		Low Response % (N = 251)	High Response % (N = 59)
Adaptive			
IL-17	7.7	7.2	9.8
IFN-γ	30.6	26.7	46.0
IL-10	17.1	12.8	34.1
IL-13	60.0	53.3	86.4
IL-5	54.8	51.7	67.2
Innate			
IL-6	15.5	0	76.2
IL-10	12.6	3.4	48.4
IL-8	41.6	27.3	97.6
TNF-α	32.6	19.8	82.7
		SEB-stimulation immune phenotype	
		Low response %	Modified Th2 response %
Adaptive			
IL-17	49.8	42.8	57.0
IFN-γ	52.6	46.7	58.6
IL-10	53.2	28.2	78.6
IL-13	52.6	14.4	91.3
IL-5	50.3	17.3	83.8

Shown are conditional probabilities estimated by latent class analysis for each of the two phenotypes. Entropy for spontaneous and SEB-stimulated was 0.895 and 0.701, respectively. Proportions of children producing low vs. high response phenotypes for spontaneous were 79.7 vs. 20.3 %, and for low vs. modified Th2 response for SEB-stimulation were 50.4 vs. 49.6 %

**Table 2:** Univariate and multivariate associations between poor hygiene exposures and immune response phenotype membership

Exposures	Spontaneous outcome (High)				SEB-stimulated outcome (Th2 modified)			
	Crude		Adjusted		Crude		Adjusted	
	OR	(95 % CI)	OR	(95 % CI)	OR	95 % CI	OR	95 % CI
Father agricultural activities (Yes vs. No)	0.81	(0.44;1.49)	0.79	(0.41;1.51)	0.57	(0.33;0.97)	0.55	(0.32;0.96)
Birth order (≥4^th^ vs. ≤3^rd^)	0.44	(0.22;0.86)	0.48	(0.24;0.95)	2.07	(1.21;3.56)	2.04	(1.18;3.52)
Any geohelminth infection (Yes vs. No)	0.24	(0.12;0.47)	0.25	(0.12;0.50)	1.68	(1.00; 2.82)	1.68	(0.98; 2.89)
Number peri-domiciliary animals (> = 4 vs. <4)	0.53	(0.19;1.44)	0.88	(0.43;1.82)	0.92	(0.54;1.56)	0.89	(0.51;1.56)

ORs adjusted for age, sex, area of residence, ethnicity, and other variables in model

The present analysis using LCA extends our previous observations of the effects of poor hygiene exposures on the production of individual cytokines to identify cytokine phenotypes associated with poor hygiene exposures. We observed previously that PBLs from children with chronic geohelminth infections [2, 4], and among those higher in the birth order [4] and living in poor conditions [3] produced higher levels of SEB-induced IL-10. Geohelminths remain a common environmental exposure in Latin American countries with the majority of studies showing prevalence greater than 20 % [6]. In the present study, we show that PBLs from children with geohelminth infections and higher in the birth order produce ‘regulated’ phenotypes of cytokine profiles both homeostatically (resulting in generalized cytokine hyporesponsiveness) and upon a maximal immune stimulation (a modified Th2 response considered to represent a tolerized Th2 response). Both types of response have been associated with chronic helminth infections in previous studies [7] and are considered to be mechanisms by which geohelminths dampen host antiparasite responses and may prevent allergy.

The study is subject to several potential limitations. Although we were able to adjust for most relevant confounders and ORs were reasonably stable to adjustment for measured confounders, we cannot exclude uncontrolled confounding by unmeasured variables. While the study was relatively large for the measurement of cytokines, the sample was limited by logistic and cost considerations and we had limited power to detect small effects or for uncommon exposures. Data for 3 of the 4 poor hygiene exposures measured were collected by parental questionnaire that may have led to some non-systematic misclassification resulting in loss of study power. Although geohelminth infections can survive in the human host for a period of years and children living in endemic areas remain infected over long periods of time through repeated reinfections, the measurement of geohelminth infections through the collection of a single stool sample, as done in the present study, cannot distinguish a chronic from a recently acquired infection in a previously uninfected child. The impact on our analysis of mixing these two different types of infection would be one of non-systematic misclassification to reduce the magnitude of the observed effects rather than a systematic bias. We were able to measure only a few innate and adaptive cytokines that have been linked with inflammatory pathology in humans and for which assays were available at the time of sample analysis. Since then other potentially important inflammatory cytokines have emerged (e.g. IL-18) and our analyses, therefore, cannot shed any light on inflammatory profiles that might include such inflammatory mediators. We felt that measurement of innate cytokines after SEB testing was unlikely to be informative given the experimental use of SEB for T cell stimulation [8]. Identifying patterns of correlated cytokines using an unbiased analytic approach such as LCA is probably preferable to the analysis of cytokines separately as if they acted in isolation.

Our data provide novel insights into how poor hygiene exposures may reduce inflammation and inflammatory diseases: through the reduction of cytokine responsiveness ‘at rest’, perhaps by reducing the state of activation of immune cells, and by blunting Th2 inflammatory responses non-specifically through the co-production of IL-10, a cytokine with potent anti-allergic effects [9]. Geohelminth infections and birth order, that have been shown to protect against allergy in numerous epidemiological studies [1, 7], were associated with similar immune phenotypes possibly because their effects are mediated through similar mechanisms. Previous studies of the effects of farming exposures on the human immune response showed that this effect is mediated through microbial exposures [10, 11]. For example, farming children exposed to high levels of endotoxin showed a down-regulation of TNF-α and IL-10 responses by SEB-stimulated PBLs [10]. Exposure to a wide diversity of microbes may be required for the induction of regulatory immunologic effects associated with geohelminth infections and birth order (representing transmission of infections from older to younger siblings) observed here. Future studies should explore the immunological pathways through which these exposures affect human immune phenotypes.

In conclusion, our observations show that specific environmental exposures affect immune response phenotypes in children living in a tropical setting and provide further insights into how poor hygiene exposures may mediate their immunologic effects on inflammatory diseases such as asthma.

## Additional files

Additional file 1: Table S1. Demographic and environmental characteristics of schoolchildren. (DOCX 18 kb)
Additional file 2: Figure S1. Relationship between immune profiles and environmental factors. The bars represent the response probabilities for each variable in respective profile. (DOC 133 kb)

## Abbreviations

Adj: Adjusted
ELISA: Enzyme-linked immunosorbent assay
HEPES: 4-(2-hydroxyethyl)-1-piperazineethanesulfonic acid
IL: Interleukin
LCA: Latent class analysis
PBLs: Peripheral blood leukocytes
RPMI 1640: Roswell Park Memorial Institute 1640
SEB: *Staphylococcus* enterotoxin B
SPT: Skin prick tests
TH2: T helper cell 2

## References

[1] Rook GA (2013). Regulation of the immune system by biodiversity from the natural environment: an ecosystem service essential to health. Proc Natl Acad Sci U S A.

[2] Figueiredo CA, Barreto ML, Rodrigues LC, Cooper PJ, Silva NB (2010). Chronic intestinal helminth infections are associated with immune hyporesponsiveness and induction of a regulatory network. Infect Immunity.

[3] Figueiredo CA, Alcântara-Neves NM, Veiga R, Amorim LD, Dattoli V, et al. Spontaneous cytokine production in children according to biological characteristics and environmental exposures. Environ Health Perspect. 2009;117:845–9. 10.1289/ehp.0800366 PMC2685851 19478971

[4] Cooper PJ, Amorim LD, Figueiredo CA, Esquivel R, Tupiza F (2015). Effects of environment on human cytokine responses. WAO J.

[5] Endara P, Vaca M, Platts-Mills TA, Workman L, Chico ME (2015). Effect of urban vs. rural residence on the association between atopy and wheeze in Latin America: findings from a case–control analysis. Clin Exp Allergy.

[6] Saboyá MI, Catalá L, Nicholls RS, Ault SK (2013). Update on the mapping of prevalence and intensity of infection for soil-transmitted helminth infections in Latin America and the Caribbean: a call for action. PLoS Negl Trop Dis.

[7] Fallon PG, Mangan NE (2007). Suppression of TH2-type allergic reactions by helminth infection. Nat Rev Immunol.

[8] Fleischer B, Schrezenmeier H (1988). T cell stimulation by staphylococcal enterotoxins. Clonally variable response and requirement for major histocompatibility complex class II molecules on accessory or target cells. J Exp Med.

[9] Figueiredo CA, Amorim LD, Alcantara-Neves NM, Matos SM, Cooper PJ (2013). Environmental conditions, immunologic phenotypes, atopy, and asthma: new evidence of how the hygiene hypothesis operates in Latin America. J Allergy Clin Immunol.

[10] Braun-Fahrländer C, Riedler J, Herz U, Eder W, Waser M (2002). Environmental exposure to endotoxin and its relation to asthma in school-age children. N Engl J Med.

[11] Ege MJ, Mayer M, Normand AC, Genuneit J, Cookson WO (2011). Exposure to environmental microorganisms and childhood asthma. NEJM.

