# Supplementary material for: Effects of poor hygiene on cytokine phenotypes in children in the tropics
Source: World Allergy Organ J. 2016 Nov 3;9(1):34. doi: 10.1186/s40413-016-0124-1 (PMC5093929; doi:10.1186/s40413-016-0124-1)
Supplement: Additional file 1: Table S1. — Demographic and environmental characteristics of schoolchildren. (DOCX 18 kb) [file 40413_2016_124_MOESM1_ESM.docx]

Additional file 1: Table S1. Demographic and environmental characteristics of schoolchildren

| Characteristics | n (%)  (n=310) |
| --- | --- |
| *Demographics* |  |
| Area of residence  Rural  Urban | 165 (53.2)  145 (46.8) |
| Sex  Males  Females | 169 (54.5)  141 (45.5) |
| Ethnicity  Afro-Ecuatorian  Mestizo | 277(89.4)  33 (10.6) |
| Age group  6-10 years  >10 years | 178 (57.4)  132 (42.6) |
| *Poor hygiene exposures*  Household overcrowding (≥ 2.7)  No  Yes | 153 (49.3)  157 (50.7) |
| Birth order  1^st^ -3^rd^  ≥4^th^ | 189 (61.0)  121 (39.0) |
| Father agricultural activities  No  Yes | 116 (37.4)  194 (62.6) |
| Geohelminth infection  No  Yes  Number of peri-domiciliary animals  <4  ≥4 | 153 (49.3)  157 (50.7)  199 (64.2)  111 (35.8) |
|  |  |
