# Supplementary material for: Effects of poor hygiene on cytokine phenotypes in children in the tropics
Source: World Allergy Organ J. 2016 Nov 3;9(1):34. doi: 10.1186/s40413-016-0124-1 (PMC5093929; doi:10.1186/s40413-016-0124-1)

Additional file 2: Figure S1. Relationship between immune profiles and environmental factors. The bars represent the response probabilities for each variable in respective profile.

**(A) Homeostasis (B) Maximal stimulus
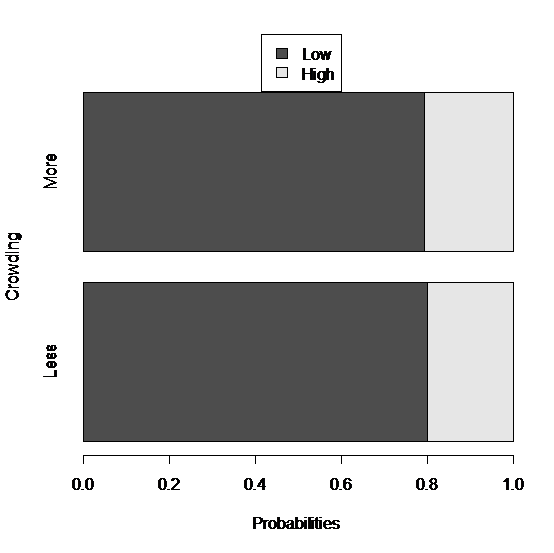

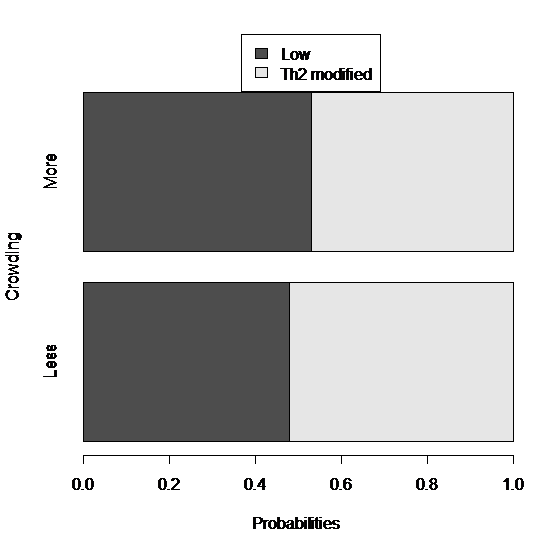
**


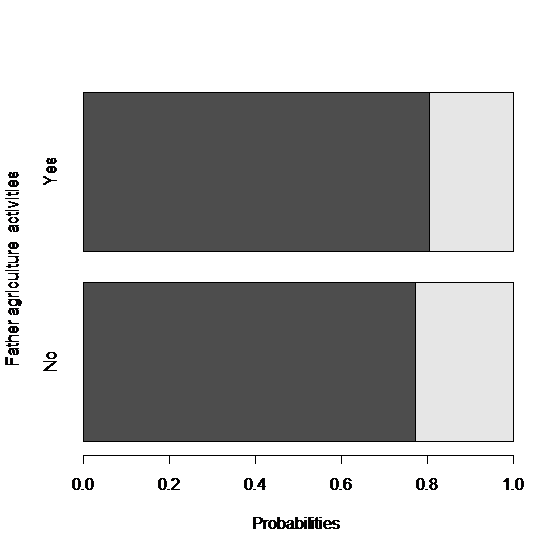

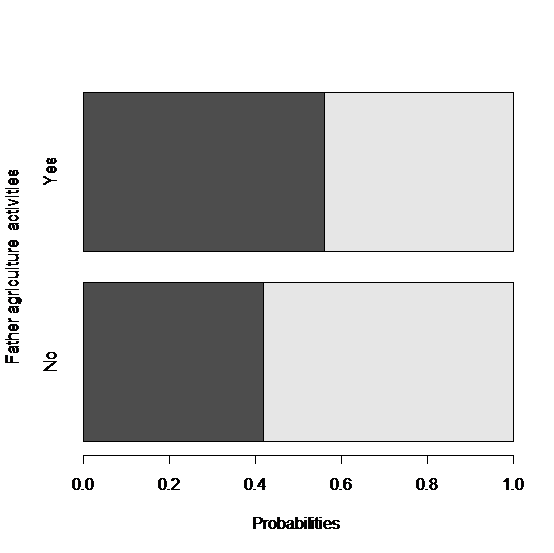


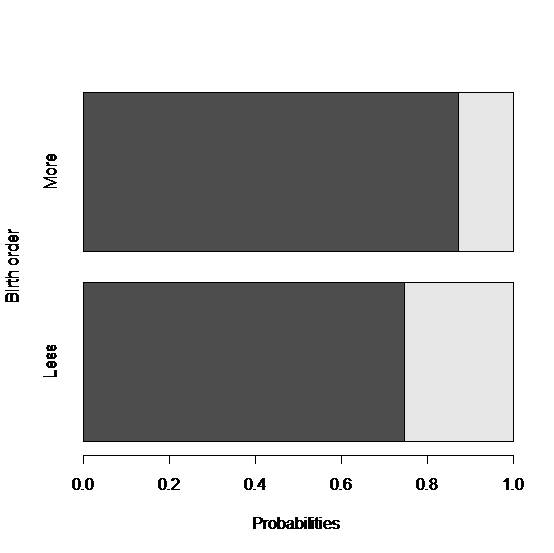

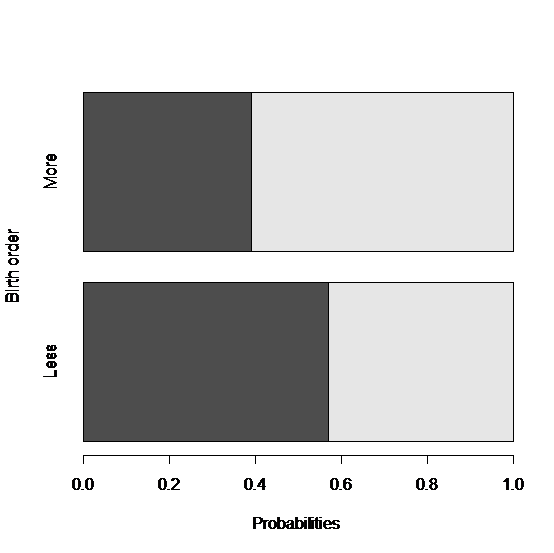


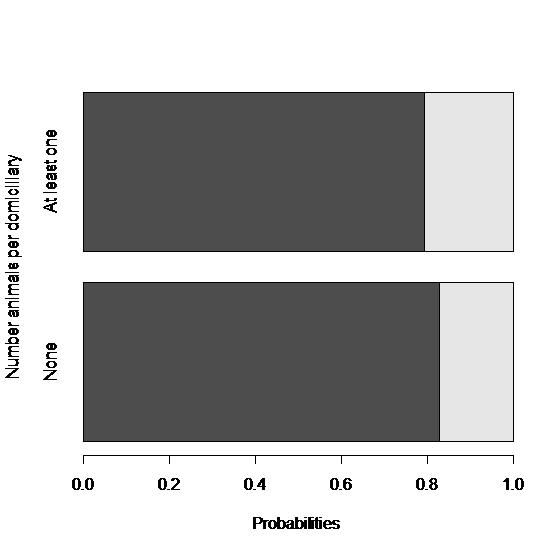

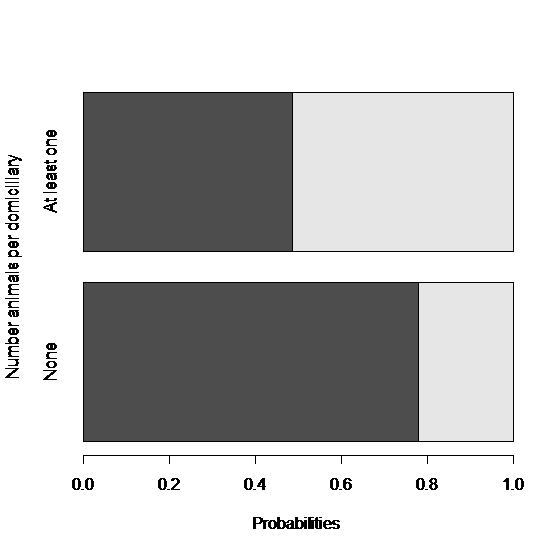


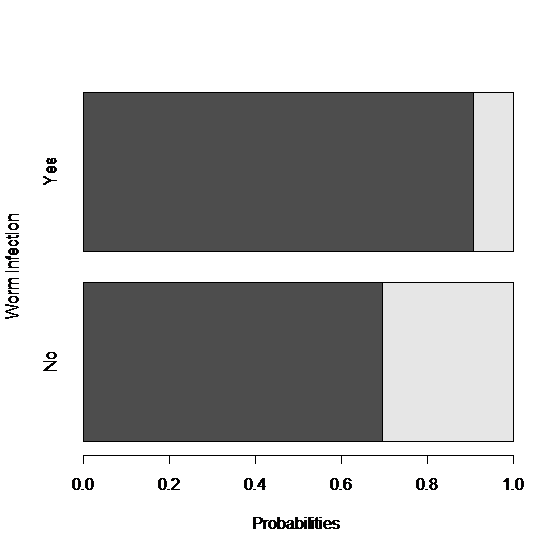

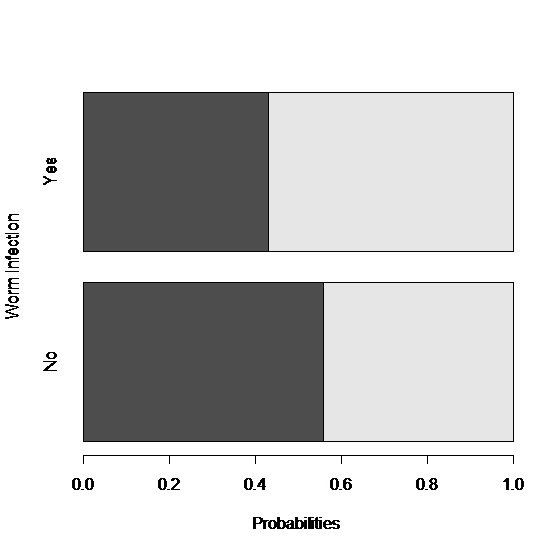

Supplement: Additional file 2: Figure S1. — Relationship between immune profiles and environmental factors. The bars represent the response probabilities for each variable in respective profile. (DOC 133 kb) [file 40413_2016_124_MOESM2_ESM.doc]
